# The UII/UT System Mediates Upregulation of Proinflammatory Cytokines through p38 MAPK and NF-κB Pathways in LPS-Stimulated Kupffer Cells

**DOI:** 10.1371/journal.pone.0121383

**Published:** 2015-03-24

**Authors:** Liang Ming Liu, Dong Yu Liang, Chang Gen Ye, Wen Juan Tu, Tong Zhu

**Affiliations:** Department of Hepatology, Songjiang Hospital Affiliated to the First People’s Hospital Shanghai Jiaotong University, Shanghai, China; Harvard Medical School, UNITED STATES

## Abstract

The urotensin II (UII)/UII receptor (UT) system is closely related to immune inflammation. In acute liver failure (ALF), the UII/UT system can promote the production and release of proinflammatory cytokines, inducing an inflammatory injury response in liver tissue. However, the mechanism by which the hepatic UII/UT system promotes proinflammatory cytokine production and release is not clear. To solve this problem, we used primary Kupffer cells (KCs) as the model system in the current study. The results showed that after lipopolysaccharide (LPS) stimulation, KCs showed significantly increased expression and release of UII/UT and proinflammatory cytokines tumor necrosis factor α (TNF-α) and interleukin 1β (IL-1β). Pretreatment with urantide, which is a UT receptor antagonist, significantly inhibited the LPS-stimulated expression and release of UII/UT, TNF-α, and IL-1β by KCs. In addition, LPS stimulation induced nuclear p38 mitogen-activated protein kinase (MAPK) protein phosphorylation and expression of the nuclear nuclear factor κB (NF-κB) p65 subunit in KCs and enhanced the binding activity of NF-κB to DNA molecules, whereas urantide pretreatment significantly inhibited the LPS-stimulated nuclear expression and activity of these molecules in KCs. Therefore, our conclusion is that the UII/UT system mediates LPS-stimulated production and release of proinflammatory cytokine by KCs, and this mediating effect at least partially relies on the inflammatory signaling pathway molecules p38 MAPK and NF-κB.

## Introduction

Acute liver failure (ALF) is a clinical syndrome characterized by severe acute liver tissue damage and rapid loss of liver function due to various factors [1]. Immune-mediated liver tissue inflammation is considered to be the major pathophysiological mechanism of ALF [2]. Among these mechanisms, the proinflammatory cytokine release cascade mediated by the innate immune response plays a central role in the pathogenesis and development of ALF [3,4]. However, the mechanisms underlying the release of immune inflammatory factors in the liver remain unexplored.

In recent years, it has been found that urotensin II (UII), a vasoactive peptide substance, is closely related to tissue damage caused by immune inflammation. The UII is a cyclic peptide that was originally isolated from bony fish tailbone and contains 11 amino acids. It was later proven that UII is widely distributed in mammals, including humans, and it is expressed in tissues and organs such as the cardiovascular system, central nervous system, lungs, kidneys, spleen, pituitary and adrenal glands, stomach, pancreas, liver, and ovaries [5–7]. The UII performs various physiological and pathological activities and can regulate endocrine as well as cardiovascular, renal, and immune functions [8]. It has been confirmed that high UII expression and secretion in inflammatory injury sites can promote chemotaxis of inflammatory cells [9], stimulate the expression of such proinflammatory cytokines as IL-6 [10], and induce the expression and secretion of cytokines and inflammatory adhesion molecules by endothelial cells [11]. Signaling via UII is primarily transduced via a specific orphan G-protein-coupled receptor, i.e., the UII receptor (often referred to as UT) [12]. Studies have shown that the expressions of UII and its UT receptor were significantly increased in the livers of patients with ALF [13]. Our recent study using an ALF mouse model revealed that UII/UT was primarily expressed in the non-parenchymal cells of the liver (i.e., Kupffer cells (KCs) and endothelial cells) and that blocking UII signaling with the UT-specific antagonist urantide could prevent LPS-induced death in ALF mice, reduce liver inflammation injury, and significantly reduce the production and release of proinflammatory cytokines in the liver, including TNF-α, IL-1β, and IFN-γ [14]. Urantide ([Pen5,DTrp7,Orn8]UII(4–11)) is a UII analog, which binds with the second extracellular loop of UT [15]. The compound competitively antagonized UII-induced effects with pKB = 8.3±0.09 and displaced [125I]UII from specific binding at UII recombinant receptor (pKi = 8.3±0.04), and has been proposed as the most potent UT antagonist so far described [16]. Therefore, it suggests that the UII/UT system can mediate the occurrence of ALF by promoting the release of proinflammatory cytokines in the liver.

It is known that intrahepatic proinflammatory cytokines are primarily derived from KCs [17]. KCs are non-parenchymal cells of the liver and account for approximately 15% of total liver cells and 80–90% of the total number of resident macrophages in vivo [18]. The KCs cover the inner liver sinusoidal wall and can act as "professional" phagocytic cells in stable or physiological conditions [19] to clear aging red blood cells, immune complexes, and intestinal bacterial products from portal circulation [18]. As an important component of the innate immunity of the body, KC activation and release of inflammatory factors are common pathophysiological mechanisms underlying liver injury due to various causes [20]. It has been confirmed that KCs can play a key role in the pathogenesis and development of immune inflammatory liver injury diseases (including ALF) by secreting various proinflammatory cytokines [20].

The expression and secretion of proinflammatory cytokines in KCs are regulated by the toll-like receptor 4 (TLR4) signaling pathway [21]. The actions of liver damage factors (such as lipopolysaccharide (LPS)) can stimulate KCs and activate the cell surface receptor TLR4, thus initiating the p38 mitogen-activated protein kinase (MAPK) and nuclear factor κB (NF-κB) signaling pathways and promoting proinflammatory cytokine expression at the transcriptional and translational levels [22]. To reveal the molecular mechanisms by which the UII/UT system mediates ALF, we further analyzed the UII/UT expression conditions of KCs under LPS stimulation based on our previous animal experiments [14], and additionally used urantide to investigate the effects of the UII/UT system on proinflammatory cytokine expression and secretion and the underlying mechanism.

## Materials and Methods

### I. Materials

#### Experimental animals

Clean healthy grade male Sprague Dawley (SD) rats with weights of 150–200 g, were provided by the Experimental Animal Center of the First People's Hospital affiliated with Shanghai Jiaotong University, and the Animal Certificate of Conformity number was SYXK (Shanghai) 2009-0086. The animals were maintained in specific pathogen free air at a temperature of 22±2°C with 12 h light and dark cycles and relative humidity of 50%. The rats had free access to water and food and were placed on 12:12 h light:dark cycles. The rats were fasted for 12 h before the experiments. Animals care and treatment were humanity and in compliance with the recommendations in the Guide for the Care and Use of Laboratory Animals of the National Institutes of Health. The protocol was approved by the Committee on the Ethics of Medical Scientific Research of the First People's Hospital, Shanghai Jiaotong University (Permit Number: 2012KY041). All surgery was performed under sodium pentobarbital anesthesia, and all efforts were made to minimize suffering.

#### Main reagents and consumables

LPS was purchased from Sigma (USA), IV collagenase was purchased from Invitrogen (USA), UII was purchased from Phoenix (USA), urantide was purchased in PEPTIDES (Japan), 1640 medium and fetal bovine serum were purchased from Gibco (USA), PCR primers were synthesized by Shanghai Sangon Biotech, the PCR reaction kit was purchased from Tiangen Biotech (Beijing) Co., the reverse transcription kit was purchased from Fermentas (Canada), the ELISA kit was purchased from Shanghai Neobioscience Technology, the real-time PCR kit was purchased from Takara Biotechnology (Dalian) Co., the nuclear extraction and electrophoretic mobility shift assay (EMSA) kit were purchased from Pierce (USA), p65 antibody was purchased from Cell Signaling (USA), and antibodies against p38 MAPK and phosphorylated p38 MAPK were purchased from Abcam (USA).

### II. Methods

#### Isolation and culture of primary KC

Rat KCs were isolated and cultured according to the method in the literature [23] with a slight improvement. In brief, rats were anesthetized via intraperitoneal injection of sodium pentobarbital (40 mg/kg) and received intraperitoneal injections of 1 mL of heparin. Disinfection was conducted by soaking in 75% alcohol. The abdomen was opened to reveal the portal vein. After ligation of the suprahepatic inferior vena cava, D-Hanks' solution at room temperature was slowly injected through the portal vein, and the hepatic inferior vena cava was cut open to allow blood and perfusion fluid to drain freely. After the liver volume showed enlargement and the color of various portions of the liver gradually turned white, the solution was switched to 0.5% IV collagenase digestion solution (prepared using Hank’s balanced salt solution (HBSS)) to continue perfusion and digestion of the liver tissue. The liver was dissected and placed in IV collagenase digestion solution (containing 0.1% pronase E and 0.005% DNase I). The liver tissue was carefully torn apart and passed through a 200-mesh filter. The filtrate was centrifuged, and the pellet was subjected to discontinuous Percoll density gradient centrifugation. The membranous cell layer in the interface between 30% Percoll and 60% Percoll was carefully collected via aspiration. Cells were washed with HBSS, added to RPMI 1640 medium (containing fetal calf serum, penicillin, and streptomycin) and resuspended. After 1–2 h of incubation, non-adherent cells were washed away to obtain purified KCs. Phagocytosis testing using ink and ED2 staining was used to identificate the purified KCs (S1 Fig.) and validate cell viability and purity. Upon cell purity > 90% and viability > 95%, cells were used for the next experiment.

#### KC treatment methods

Cells were seeded in 6-well plates with 4 × 10^6^ cells per well. After 24 h of incubation, cells were washed three times with phosphate buffered solution (PBS), and 500 μL of serum-free culture medium was added to each well, followed by pretreatment using UII or urantide solution (both were prepared in PBS). The UII final concentration was 100 nM, that of urantide was 20 nM, and the dosage and usage were applied as previously described [24–26] with minor modifications. After half an hour, LPS solution (20 μg/mL, prepared in PBS) was used to stimulate the cells. The final volume in each well was 515 μL. Six hours after LPS stimulation, cells and the culture supernatants were collected for use in subsequent experiments. The experiment was repeated six times.

#### RT-PCR detection

Cultured KCs were treated with Trizol to extract total cellular RNA. Extraction of total RNA was performed according to the manual. Total RNA was used as the template for the synthesis of first-strand cDNA. The resulting cDNA was used as the template, Taq DNA polymerase and the downstream and upstream primers for the target genes were added, and PCR amplification was performed according to the manual. Primer design was carried out using Primer Premier 6.0 software (PREMIER Biosoft International), and the primer sequences and product length used in gene detection are shown in Table 1. The reaction conditions were as follows: denaturation at 94°C for 5 min; 32 cycles of 94°C for 1 min, 51°C for 45 s, and 72°C for 45 s; and extension at 72°C for 10 min. The resulting PCR product was electrophoresed in 1.5% agarose gel, and β-actin was used as the internal reference. Electrophoresis results were measured by BIO-RAD Quantity-One 4.7 image analysis software to calculate the relative gray scales of the detected genes as compared with that of β-actin.

**Table 1:** Primer sequences and product lengths used in gene detection by RT-PCR

Genes	Primer sequences (5′→3′)		Product length
TNF-α	Sense	GGCGTGGAGCTGAGAGATAAC	92bp
	Antisense	GGTGTGGGTGAGGAGCACAT	
IL-1β	Sense	TCCCCAGCCCTTTTGTTGA	120bp
	Antisense	TTAGAACCAAATGTGGCCGTG	
β-actin	Sense	TGTTACAGGAAGTCCCTTGCC	101bp
	Antisense	AATGCTATCACCTCCCCTGTG	

#### Real-time PCR detection

Extractions of total RNA and first-strand cDNA synthesis were performed as described in the RT-PCR methods. Primers were designed with Primer Premier 6.0 software, and the sequences and product lengths are shown in Table 2. Real-time PCR was performed according to kit instructions. The reaction system was supplemented with 2 × Premix Ex Taq^TM^ II, sense and antisense strand primers, ROX Reference Dye II, and DNA templates and was amplified in the ABI 7500 instrument using the following two-step method: Step One: 50°C for 2 min, 1 cycle; Step Two: 95°C for 5 min, 40 cycles. Glyceraldehyde 3-phosphate dehydrogenase (GAPDH) was used as the internal reference.

**Table 2:** Primer sequences and product lengths used in gene detection by real-time PCR

Genes	Primer sequences (5′→3′)		Product length
UII	Sense	5' GGGATGGCAGCCCTAAACACAG 3'	109bp
	Antisense	5' TCCGAGCAGAAGTGACGCAGAG 3'	
UT	Sense	5' GCATCGTGCTGCTCTTCTGG 3'	123bp
	Antisense	5' GGCAGGTGGTCAGGTAGTTG 3'	
GAPDH	Sense	5' CACCCACTCCTCCACCTTTG 3'	110bp
	Antisense	5' CCACCACCCTGTTGCTGTAG 3'	

#### Enzyme-linked immunoassay (ELISA)

The TNF-α and IL-1β in the cell supernatant were measured by double-antibody sandwich ELISA, and the procedures were performed in accordance with the kit instructions. In brief, the procedures are described as follows: samples or standards (100 uL/well) were added, followed by 90 min of incubation at 36°C; the plates were washed five times, and the biotinylated antibody solution (100 μL/well) was added, followed by 60 min of incubation at 36°C; the plates were washed five times and the enzyme conjugate working solution (100 uL/well) was added, followed by 30 min of incubation at 36°C in the dark; the plates were washed five times and the substrate (100 uL/well) was added, followed by 15 min of incubation at 36°C in the dark; the stop solution (100 uL/well) was added, and the OD450 values were measured after thorough mixing. The LPS or PBS treatment was applied to triplicate wells, and the results are presented as the mean of the triplicates.

#### Extraction of nuclear protein

The procedures were conducted according to the instructions in the Pierce nuclear protein extraction kit. In brief, the following procedures were performed: cells were trypsinized and collected via centrifugation at 500 g for 5 min. The collected cells were washed twice with pre-chilled PBS, resuspended in CER I buffer at a 1:10 ratio by volume, and shaken vigorously for a few seconds for thorough re-suspension, followed by incubation on ice for 10 min. Ice-cold CER II buffer was added and thoroughly mixed, followed by incubation on ice for 1 min. The suspension was centrifuged at 16000 g for 5 min, the supernatant was discarded, and the pellet contained the nuclei. An equal volume of NER ice bath buffer was added to the pellet and mixed well, followed by incubation on ice for 40 min and centrifugation at 4°C and 16,000 g for 10 min. The supernatant represented the nuclear extract. UV spectrophotometry was used to determine the nuclear protein concentration, and the extract was subsequently aliquoted and stored at −70°C.

#### Western-blot analysis

A total of 50 μg of protein was collected, boiled in Laemmli buffer for 10 min, and subjected to 10% sodium dodecyl sulfate/polyacrylamide (SDS-PAGE) electrophoresis, followed by transfer to the PVDF membrane. The PVDF membrane bound with proteins was blocked in 5% fat-free milk overnight at 4°C. After blocking, appropriate amounts of antibodies recognizing p65, p38, p-p38, and the internal reference actin were added, followed by incubation at room temperature for 2 h. The membrane was washed three times with 0.1% phosphate buffered saline Tween-20 (PBST) with 5 min per wash. Horseradish-peroxidase-labeled secondary antibodies were added, followed by incubation at room temperature for 1 h. The membrane was subsequently washed three times with 0.1% PBST with 5 min per wash. The results were detected with the ECL-Plus chemiluminescent detection kit, followed by X-ray film exposure. The results were observed after development and fixation of the film.

#### Method for biotin-labeled DNA probes

Two complementary NF-κB binding DNA probes were synthesized (by Shanghai Sangon Biotech). The sequences were 5'-AGTTGAGGGGACTTTCCCAGGC-3' and 3'-TCAACTCCCCTGAAAGGGTCCG-5'. Biotin labeling of the two DNA sequences was performed according to kit instructions. In brief, the following procedures were performed: the single-stranded probe for labeling was diluted with water to 1 μM and placed on ice; 25 μl of ultra pure water was added to the centrifuge tubes together with 10 μL of 5 × TdT reaction buffer, 5 μL of unlabeled oligonucleotide probes, 5 μL of Biotin-11-UTP (5 μM), and 5 μl of TdT (2 U/μL), followed by incubation at 37° C for 30 min; the labeling reaction was terminated by adding 2.5 μL of 0.2 M EDTA; 50 μL of chloroform were added to the tubes with isoamyl alcohol (24:1) and centrifuged at 13,000 g after mild vortexing for 2 min; the upper liquid phase was recovered and transferred; equal volumes of the labeled single-stranded sense and an antisense DNA probes were mixed, annealing buffer was added, and the tubes were incubated at 95°C for 2 min, followed by slow cooling to Tm and maintenance at Tm for 30 min. The products were stored at −20°C.

#### Electrophoretic mobility shift assay (EMSA)

After the protein concentrations were measured, the extracted nuclear protein components were used for EMSA, according to the literature [27] and the kit manual. The reaction tubes were supplemented with 2 μL of 10 × binding buffer, 1 μL of 50% glycerol, 1 μL of 100 mM MgCl_2_, 1 μL of 1 μg/μL poly (dI • dC), 1 μL of 1% NP-40, 1 μL of 1 M KCl, 1 μL of 200 mM EDTA, 1 μL of labeled probe (10 um), and 3 μl of nuclear protein (1.5 μg/μL), and ultrapure water was added at the end to reach a final volume of 20 μL; the binding reaction lasted for 20 min under room temperature; the reaction products were separated using non-denaturing polyacrylamide gel electrophoresis; 0.5×TBE solution was used as the membrane transfer solution to transfer the probes, proteins, and probe-protein complexes in the EMSA gel to a nylon membrane; the nylon membrane was cross-linked under 254-nm ultraviolet light in UV cross-linking equipment, and the biotin-labeled probes were detected by a chemiluminescent method followed by X-ray film exposure; Image J software was used to measure the gray-scale value of each band. The average gray-scale value of the bands in the normal control group was used as the standard value of "1", and the relative value of each group was calculated to represent the relative DNA binding activity of the nuclear transcription factor NF-κB.

#### Statistical analysis

The results were expressed as (mean ± SD) and intergroup comparison was performed using one-way analysis of variance (ANOVA). The SPSS13.0 statistical software was used for statistical analysis, and *P* <0.05 indicated a statistically significant difference.

## Results

### Expression of UII and UT in primary KCs with or without LPS

**Fig 1:**
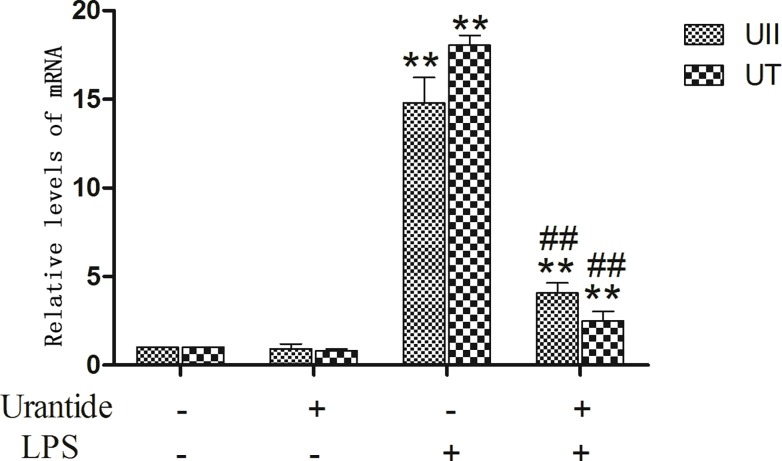
mRNA expression of UII and UT in primary KCs. Relative expression levels of UII and UTR mRNA were detected in the cells after normalization to GAPDH through real-time PCR. Data represent means ± SD (n = 6). *P<0.05 and ** *P*<0.01 versus control cells [urantide(-)LPS(-)]; #*P*<0.05 and ##*P*<0.01 versus LPS-stimulated cells [urantide(-)LPS(+)].

**Fig 2:**
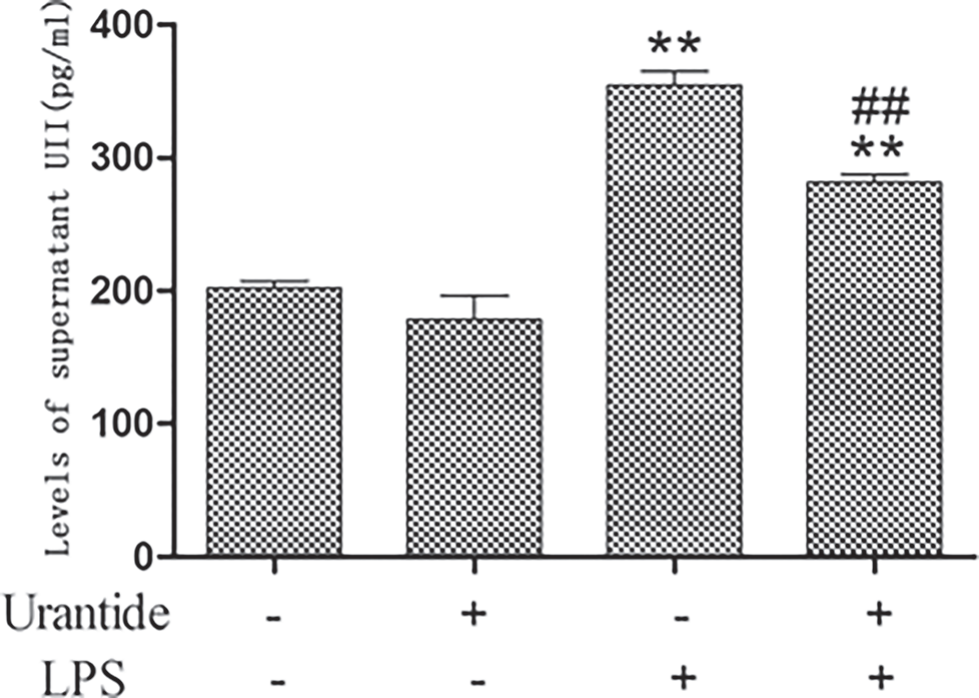
Levels of UII secretion in culture supernatant of primary KCs. The supernatant were assayed for UII secretion via ELISA. Data represent six independent studies. Values are mean ± SD (n = 6). *P<0.05 and ** *P*<0.01 versus control cells [urantide(-)LPS(-)]; #*P*<0.05 and ##*P*<0.01 versus LPS-stimulated cells [urantide(-)LPS(+)].

Our previous studies found that UII/UT expression in rat liver tissue with LPS/D-GalN-induced ALF was significantly increased [14]. To understand whether the UII/UT system is related to the liver immune inflammation, we studied UII/UT expression in KCs, which are the main hepatic innate immune cells. Our study showed that after LPS stimulation, UII and UT mRNA expression levels were significantly increased in KCs (all *P*<0.01 versus control cells), whereas pretreatment using the UT antagonists urantide significantly inhibited LPS-induced UII/UT mRNA expression (all *P*<0.01 versus LPS-stimulated cells) (Fig. 1). In addition, we also measured the UII peptide secretion levels in KC culture supernatant. We observed that after LPS stimulation, the UII peptide level in KC culture supernatant was significantly increased (*P*<0.01 versus control cells), whereas this level was significantly reduced after urantide pretreatment (*P*<0.01 versus LPS-stimulated cells) (Fig. 2). This result suggests that LPS stimulation can induce the expression and secretion of UII by KCs and promote UT expression in cells and that an autocrine and/or paracrine regulation mechanism might exist for the expression of the UII/UT system in cells.

### Urantide treatment blocks LPS-mediated stimulation of TNF-α and IL-1β in primary KCs

**Fig 3:**
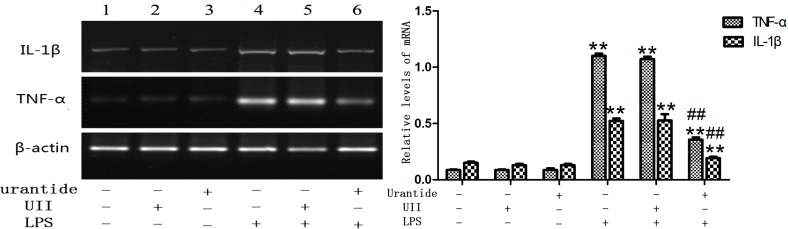
TNF-α and IL-1β mRNA expression in primary KCs. Left panel shows a representative ethidium bromide-stained gel of RT-PCR products. Relative expression levels of these cytokines in KCs are shown in the right panel after normalization to β-actin. Lane 1: UII(-)urantide(-)LPS(-); Lane 2: UII(+)urantide(-)LPS(-); Lane 3: UII(-)urantide(+)LPS(-); Lane 4: UII(-)urantide(-)LPS(+); Lane 5: UII(+)urantide(-)LPS(+); Lane 6: UII(-)urantide(+)LPS(+).Bars represent means ± SD (n = 6). *P<0.05 and ** *P*<0.01 versus control cells [UII(-)urantide(-)LPS(-)]; #*P*<0.05 and ##*P*<0.01 versus LPS-stimulated cells [UII(-)urantide(-)LPS(+)]

**Fig 4:**
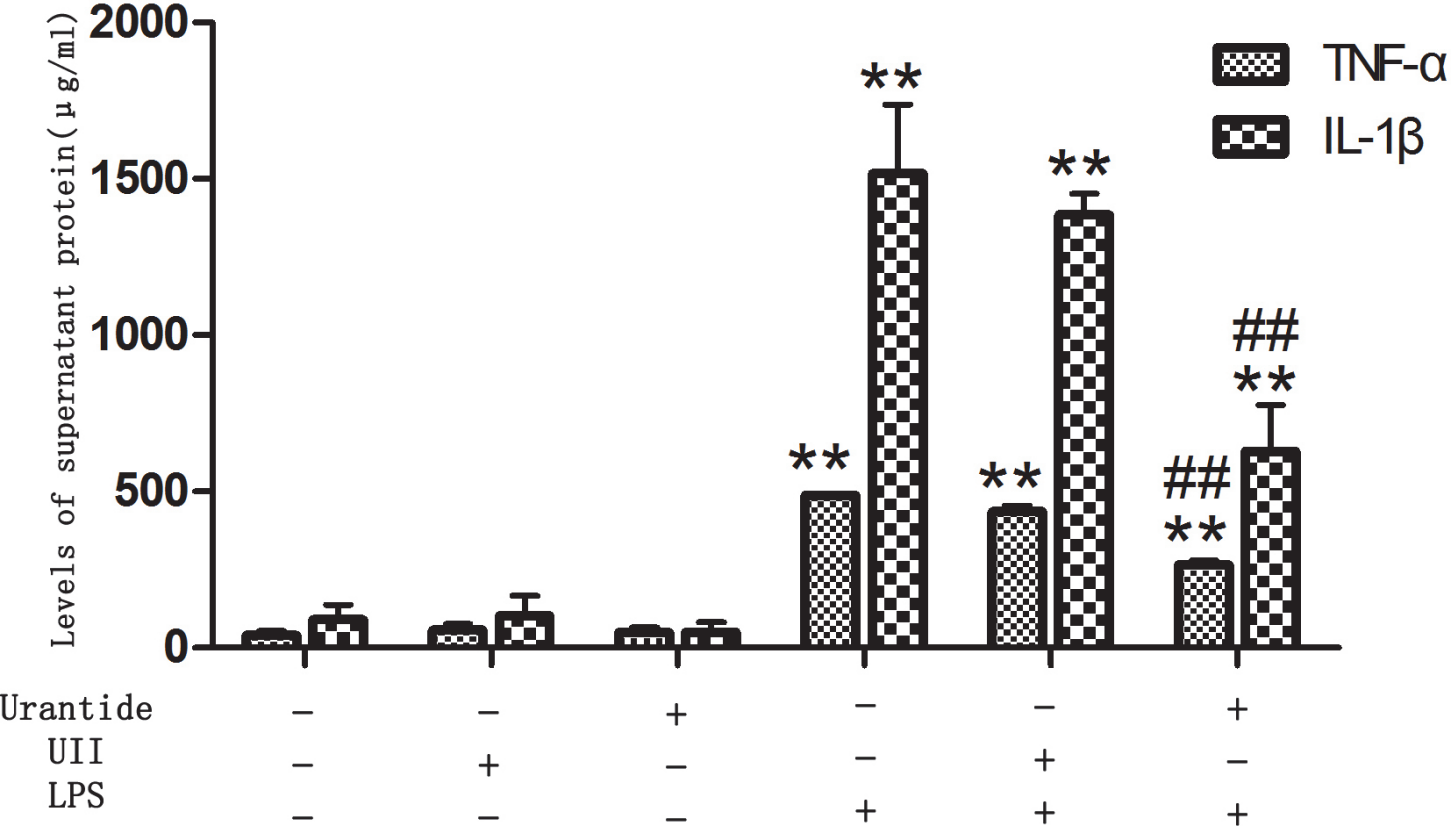
Levels of TNF-α and IL-1β in culture supernatant of KCs through ELISA. Bars represent means ± SD (n = 6). *P<0.05 and ** *P*<0.01 versus control cells [UII(-)urantide(-)LPS(-)]; #*P*<0.05 and ##*P*<0.01 versus LPS-stimulated cells [UII(-)urantide(-)LPS(+)]

To investigate the role of UII/UT system in hepatic inflammation, we studied the expression and secretion of the proinflammatory cytokines TNF-α and IL-1β by KCs. We found that after LPS stimulation, the TNF-α and IL-1β mRNA expression levels of KCs were significantly increased (all *P*<0.01 versus control cells), whereas the use of urantide pretreatment significantly inhibited LPS-induced TNF-α and IL-1β mRNA expression (all *P*<0.01 versus LPS-stimulated cells) (Fig. 3). In addition, we also detected the levels of secreted TNF-α and IL-1β proteins in KC culture supernatants. The results showed that the TNF-α and IL-1β levels in the LPS-stimulated KC culture supernatant were significantly increased (all *P*<0.01 versus control cells), and the levels were significantly decreased after urantide pretreatment (all *P*<0.01 versus LPS-stimulated cells) (Fig. 4). This result suggests that urantide can inhibit LPS-stimulated TNF-α and IL-1β expression and secretion in KCs, indicating that LPS-stimulated KC inflammatory activation depends on the UII/UT signaling system. However, KCs treated with UII alone or treated with only urantide did not show statistical differences in comparison with control cells in TNF-α and IL-1β expression and secretion.

### Urantide treatment blocks LPS-induced p38 MAPK upregulation in primary KCs

**Fig 5:**
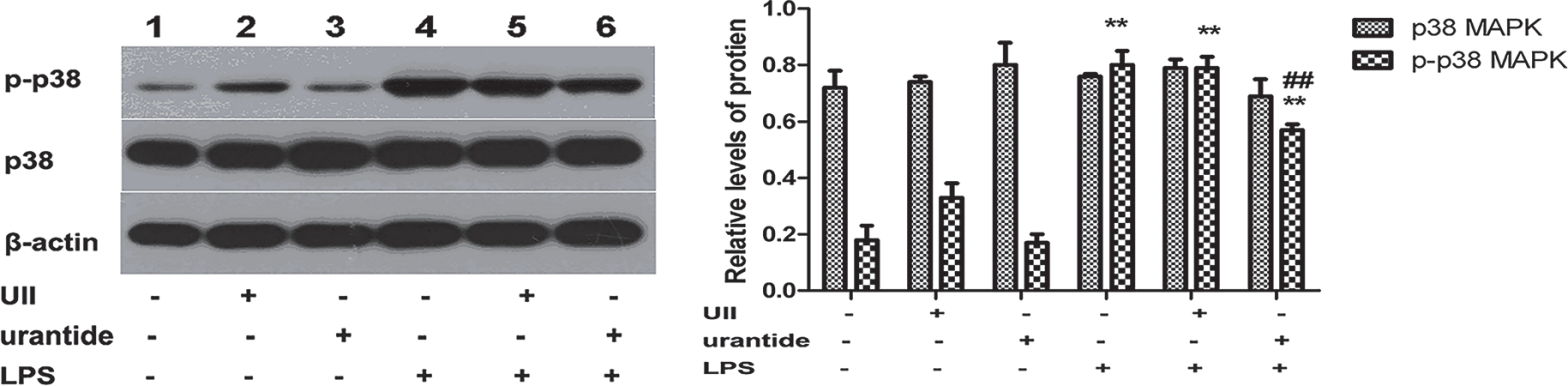
Expression of nuclear p38 MAPK and phosphorylated p38 MAPK (p-p38 MAPK) in various groups of primary KCs. Left panel shows a representative picture of Western blot, and right shows the relative levels of p38 MAPK and phospho-p38 MAPK protein in KCs after normalization to β-actin. Lane 1: UII(-)urantide(-)LPS(-); Lane 2: UII(+)urantide(-)LPS(-); Lane 3: UII(-)urantide(+)LPS(-); Lane 4: UII(-)urantide(-)LPS(+); Lane 5: UII(+)urantide(-)LPS(+); Lane 6: UII(-)urantide(+)LPS(+). Bars represent means ± SD (n = 6). *P<0.05 and ** *P*<0.01 versus control cells [UII(-)urantide(-)LPS(-)]; #*P*<0.05 and ##*P*<0.01 versus LPS-stimulated cells [UII(-)urantide(-)LPS(+)]

To study the mechanism by which the UII/UT system affects TNF-α and IL-1β expression, we examined the expression of nuclear p38 MAPK protein in KCs. The results showed no statistically significant differences in the expression level of nuclear p38 MAPK protein among groups. We next measured the phosphorylation levels of nuclear p38 MAPK protein (p-p38 MAPK) and found that KCs treated with UII alone or treated with urantide did not show statistically significant differences in nuclear p-p38 MAPK expression, whereas LPS treatment significantly increased the nuclear p-p38 MAPK levels (*P*<0.01 versus control cells). Pretreatment with urantide significantly inhibited the LPS-stimulated expression of p-p38 MAPK in KCs (*P*<0.01 versus LPS-stimulated cells) (Fig. 5).

### Urantide treatment blocks LPS-induced p65 upregulation in primary KCs

**Fig 6:**
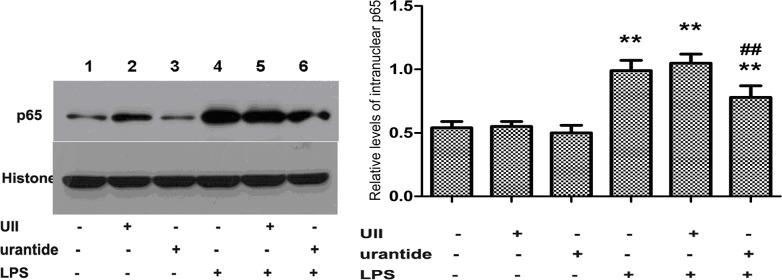
Expression of the nucleus NF-κB p65 subunit in various groups of primary KCs. Left panel shows a representative picture of Western blot, and right shows the relative levels of p65 protein in KCs after normalization to histone. Lane 1: UII(-)urantide(-)LPS(-); Lane 2: UII(+)urantide(-)LPS(-); Lane 3: UII(-)urantide(+)LPS(-); Lane 4: UII(-)urantide(-)LPS(+); Lane 5: UII(+)urantide(-)LPS(+); Lane 6: UII(-)urantide(+)LPS(+). Bars represent means ± SD (n = 6). *P<0.05 and ** *P*<0.01 versus control cells [UII(-)urantide(-)LPS(-)]; #*P*<0.05 and ##*P*<0.01 versus LPS-stimulated cells [UII(-)urantide(-)LPS(+)]

We also examined the expression of nuclear NF-κB p65 protein in KCs. The results showed that KCs treated with UII alone or treated with urantide did not show statistically significant differences in nuclear p65 protein compared with that of the normal control group, whereas the level of nuclear p65 protein was significantly increased after LPS treatment (*P*<0.01 versus control cells). Pretreatment with urantide significantly inhibited the LPS-stimulated p65 protein expression in KCs (*P*<0.01 versus LPS-stimulated cells) (Fig. 6).

### Urantide treatment blocks LPS-induced DNA-binding activity of NF-κB in primary KCs

**Fig 7:**
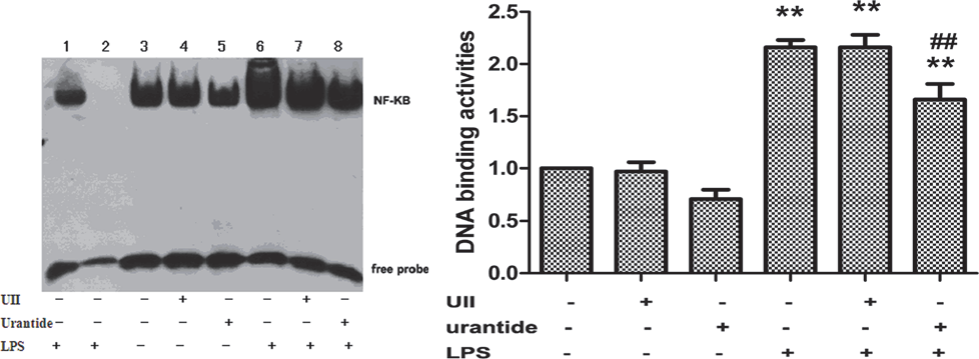
DNA-binding activity of NF-κB in nuclear protein extracts in various groups of primary KCs. DNA-binding activity of NF-κB was analyzed by EMSA (left). Lane 3, 4, 5, 6, 7 and 8 are target reactions (nuclear extract + biotin-DNA probe). Lane 3: UII(-)urantide(-)LPS(-); Lane 4: UII(+)urantide(-)LPS(-); Lane 5: UII(-)urantide(+)LPS(-); Lane 6: UII(-)urantide(-)LPS(+); Lane 7: UII(+)urantide(-)LPS(+); Lane 8: UII(-)urantide(+)LPS(+). Lane 1 and 2 are control reactions. Lane 1: cold competitive reaction of mutation DNA probe (nuclear extract + biotin-DNA + 200-fold molar excess of unlabeled mutation DNA); lane 2: cold competitive reaction of DNA probe (nuclear extract + biotin-DNA + 200-fold molar excess of unlabeled DNA). The bands corresponding to NF-κB were quantitated by densitometry (right). Bars represent means ± SD (n = 6). *P<0.05 and ** *P*<0.01 versus control cells; #*P*<0.05 and ##*P*<0.01 versus LPS-stimulated cells

We further examined the DNA binding activity of nuclear NF-κB in KCs. The results showed that KCs treated with UII alone or with urantide did not show statistically significant differences in DNA binding activity of nuclear NF-κB compared with that of the normal control group, whereas LPS treatment led to significantly increased DNA binding affinity of NF-κB (*P*<0.01 versus control cells). Pretreatment with urantide significantly inhibited the LPS-stimulated NF-κB DNA binding affinity in KCs (*P*<0.01 versus LPS-stimulated cells) (Fig. 7).

## Discussion

Our previous animal studies found that urantide, a specific antagonist that targets the UII receptor (UT), can inhibit TNF-α, IL-1β, and IFN-γ expression and secretion in mouse liver tissues [14]. The biological mechanisms underlying this phenomenon are not clear. In this study, we used primary KCs to investigate the relationship between the expression of UII/UT and proinflammatory cytokines.

In this study, we found that after LPS stimulation, the expression levels of UII/UT as well as TNF-α and IL-1β genes were significantly increased in KCs. In addition, the secretion levels of UII polypeptide and TNF-α and IL-1β proteins in cell culture supernatant were also significantly increased. This result indicates that LPS stimulation not only induces the expression of proinflammatory cytokines or inflammatory activation of KCs but also promotes signaling transduction of the UII/UT system. To explore the relationship between the UII/UT signaling system and KC-inflammatory activation, we evaluated the impact of urantide application on the expression and release of proinflammatory cytokines. We found that urantide pretreatment significantly inhibited LPS-stimulated expression and release of the proinflammatory cytokines TNF-α and IL-1β by KCs. This finding suggests that the inflammatory activation effects of LPS stimulation on KCs are dependent on the UII/UT system. In addition, urantide pretreatment also significantly inhibited the expression of UII and its receptor UT. We can infer that LPS stimulation may induce UII autocrine and paracrine regulatory mechanisms and thus facilitate the expression and secretion of proinflammatory cytokines. Therefore, UII peptide may have inflammatory hormone-like effects. Past studies have shown that the proinflammatory cytokine IFN-γ can induce the upregulation of UT expression [28], and conversely, UII upregulation also can cause the expression of the proinflammatory cytokine IL-6 [10]. This result suggests that in inflammatory conditions, mutual promoting and stimulating effects may occur between the UII/UT system and proinflammatory cytokine expression, i.e., positive feedback effects. In the liver, such a positive feedback control mechanism of KCs might aggravate the liver immune inflammatory injury response, resulting in a vicious cycle.

To further explore the inflammatory activation mechanism by which the UII/UT system mediates LPS stimulation of KCs, we investigated the expression and activity conditions of two key molecules of intracellular inflammatory signaling pathways, namely, p38 MAPK and NF-κB. It is known that the inflammation stimulating signals of LPS on KCs are primarily transduced via the TLR4 pathway. The TLR4 is the cell surface receptor of LPS. After TLR4 binds to LPS, it can form further complexes with MD-2 and activate the downstream p38 MAPK and NF-κB pathways through the adapter protein MyD88 [22]. Among these pathways, p38 MAPK is considered to be the center signaling pathway molecule in inflammatory activation mechanisms [29]. In this study, we found that urantide application significantly inhibited LPS-induced p38 MAPK phosphorylation. The p38 MAPK is a MAPK family member and is activated by phosphorylation in the nucleus, thereby inducing the transcription and expression of pre-inflammatory cytokines [30]. This finding suggests that urantide plays an anti-inflammatory role by inhibiting the activation of p38 MAPK and also shows that LPS-mediated activation of p38 MAPK requires the UII/UT signal system. Thus, KC inflammatory response induced by LPS stimulation is achieved through the activation of the inflammatory signaling pathway molecule p38 MAPK by the UII/UT system.

In addition, the NF-κB signaling pathway also plays a crucial role in the innate immune response and inflammation reaction [31]. In the cytoplasm, NF-κB primarily exists in the form of p50/p65 heterodimer, and inflammatory stimuli can activate NF-κB and cause nuclear transfer of the p65 subunit. In the nucleus, the p65 subunit binds with DNA molecules and induces the transcription and expression of various inflammation-related genes, i.e., the proinflammatory cytokines of TNF-α and IL-1β [32]. Our experiments showed that urantide significantly inhibited the LPS-stimulated NF-κB p65 subunit expression in KCs and the DNA binding activity of p65. This finding suggests that the inflammatory activation effect of the UII/UT system in LPS stimulation of KCs is also dependent on the NF-κB pathway.

In this study, we also found that UII treatment alone in the absence of LPS had no significant stimulatory effect on proinflammation production (such as TNF-α and IL-1β), p38 MAPK phosphorylation and NF-κB expression and activity of KCs, and UII and LPS together did not show additive effects. The reason for this observation may be related to the low UT expression level when KCs are in a non-activated state (or resting state). Our research shows that prior to LPS stimulation, KCs have low expression levels of UII and its UT receptor. At a low UT expression levels, it may be difficult to produce significant stimulating effects using a relatively low dose of exogenous UII. In addition, it has been shown that after inflammatory stimulation of cells, not only do cell membranes show UT expression [13] but large quantities of UT also occur within the nuclear membrane [33]. These results suggest that UII/UT signaling uses intracellular pathways. The observation that exogenous UII fails to show the originally envisaged stimulating effect of cells is not only related to the low expression of receptors but also related to the fact that it is difficult for exogenous UII to enter cells, thus lacking the effect mediated by the intracellular pathways of this system.

In conclusion, our study confirmed that the roles of the UII/UT signaling system in mediating LPS-stimulated inflammatory activation of KCs rely on or at least partially rely on the inflammatory signaling pathway molecules of p38 MAPK and NF-κB. The results of this study aid in clarifying the immune inflammatory/injury mechanism of the UII/UT system in ALF and provide an experimental foundation for the selection of ALF anti-inflammatory drug targets in the future.

## Supporting Information

S1 Fig Microscopic manifestion and identification of primary KCs. A, Morphology of KCs in light microscopy after 4 h of culture. The cells were isolated from rat liver tissue through liquid infusion, collagenase digestion and density gradient centrifugation; B, Ink phagocytosis test, showing many phagocytosed ink droplets in KC cytoplasm; C and D, ED2 staining, showing the positive cells with yellowish brown in color (C) and the control cells without staining (D).
(TIF)

S1 File Identification of primary KCs. (DOC)
