# Supplementary figures and images for: The UII/UT System Mediates Upregulation of Proinflammatory Cytokines through p38 MAPK and NF-κB Pathways in LPS-Stimulated Kupffer Cells
Source: PLoS One. 2015 Mar 24;10(3):e0121383. doi: 10.1371/journal.pone.0121383 (PMC4372515; doi:10.1371/journal.pone.0121383)

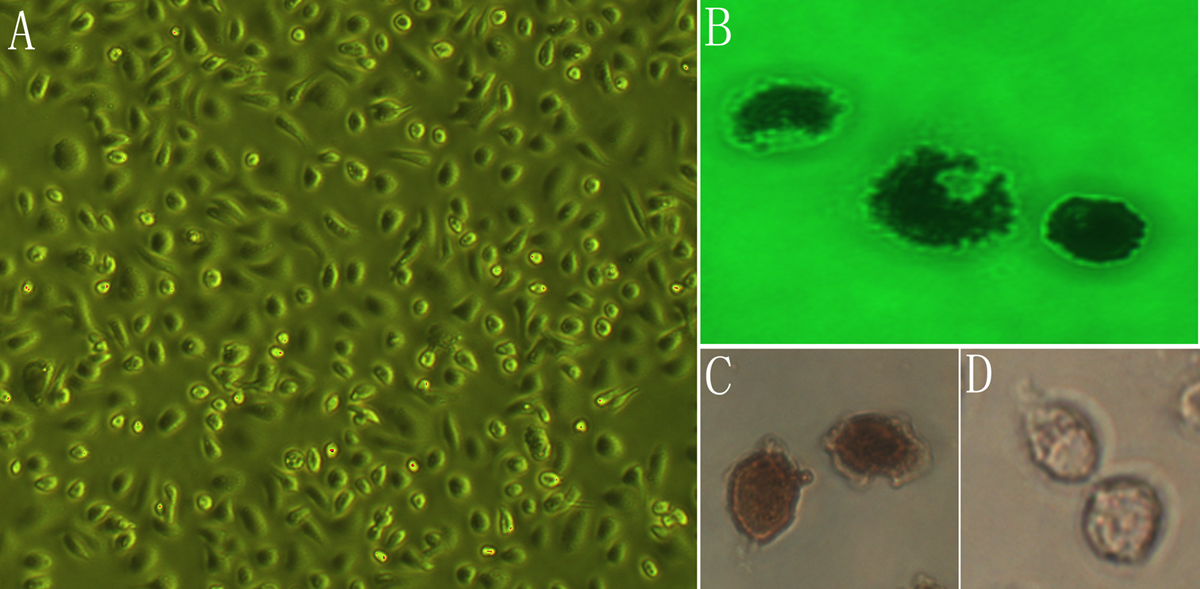

Supplement: S1 Fig — A, Morphology of KCs in light microscopy after 4 h of culture. The cells were isolated from rat liver tissue through liquid infusion, collagenase digestion and density gradient centrifugation; B, Ink phagocytosis test, showing many phagocytosed ink droplets in KC cytoplasm; C and D, ED2 staining, showing the positive cells with yellowish brown in color (C) and the control cells without staining (D). (TIF) [file pone.0121383.s001.tif]
