# Supplementary material for: The UII/UT System Mediates Upregulation of Proinflammatory Cytokines through p38 MAPK and NF-κB Pathways in LPS-Stimulated Kupffer Cells
Source: PLoS One. 2015 Mar 24;10(3):e0121383. doi: 10.1371/journal.pone.0121383 (PMC4372515; doi:10.1371/journal.pone.0121383)
Supplement: S1 File — (DOC) [file pone.0121383.s002.doc]

**S1_file. Identification of primary KCs**

Isolated primary KC might show more significant changes in morphology as the culture time is extended. This phenomenon is significantly different from that of primary hepatic parenchymal cells. After initial isolation, KC appeared under a microscope as highly refractive spheres. After 30 min of culture, KC gradually became adherent and oblate in shape. After 4 h of culture, KC became firmly adherent and displayed irregular shapes, with a subset exhibiting extended pseudopodia (Fig. A). With the extension of culture time, the cells sent out increasing amounts of pseudopodia. After 24 h, the cells showed fully extended morphology and were generally the same size, but the cell morphology was irregular, often appearing as a star, polygon, or cross in shape. KC has a strong phagocytic ability against foreign bodies. Therefore, KC can be identified by the ink phagocytosis test. Fig. B shows that the KC cytoplasm contains many phagocytosed ink droplets. The KCs contain a specific CD163 molecule, and cells appear to be yellowish brown in color after ED2 staining. Fig. C shows KCs positive for ED2 staining.
